# Neoplastic Lesions of the Mesentery: A Case Series of Rare Pathologies

**DOI:** 10.7759/cureus.103834

**Published:** 2026-02-18

**Authors:** Marcia Mejia, Mateo Zapata, Sebastian Acevedo Ramirez, Juan Muñoz

**Affiliations:** 1 Radiology, Universidad de Antioquia, Medellín, COL; 2 General Medicine, Clínica el Rosario, Medellín, COL; 3 Radiology, Hospital Pablo Tobón Uribe, Medellín, COL

**Keywords:** diagnostic imaging, mesentery, neoplasm metastasis, neoplasms, tomography

## Abstract

Mesenteric neoplasms are rare and frequently unconsidered in diagnosis. They include primary and secondary neoplasms, as well as some non-neoplastic conditions that mimic tumors. Diagnosis is often challenging, though certain clinical and radiologic features can aid early identification. Here, we present a series of histopathologically confirmed mesenteric neoplastic and non-neoplastic lesions, highlighting representative imaging features and reviewing relevant literature, with emphasis on tomographic findings.

## Introduction

The mesentery is made up of a double layer of peritoneum and is subdivided into the true mesentery and the specialized mesentery. It contains multiple structures inside and its function is to connect the small intestine to the posterior wall of the abdomen, as well as connecting multiple organs to each other [1].

Mesenteric neoplasms are rare lesions and are mostly secondary in nature, mainly because their close relationship with vascular, lymphatic, and nervous structures and multiple intra-abdominal organs makes them an important route for tumor dissemination [2]. Secondary neoplastic involvement of the mesentery occurs through different routes that can occur simultaneously and include: direct, lymphatic, hematogenous, and peritoneal spread, giving rise to different neoplasms that may have a similar appearance and originate in intra- and extra-abdominal organs. However, there are also primary mesenteric neoplasms (some of which are benign) and inflammatory or infectious lesions that mimic neoplasms, but these are rare conditions and are usually very unlikely to be suspected [3]. The symptoms of primary, secondary, and inflammatory lesions are non-specific and include abdominal pain, weight loss, palpable masses, diarrhea, among others [1].

The differential diagnosis between these conditions is difficult and usually requires histopathological analysis for confirmation. However, diagnostic imaging (especially tomography) plays a fundamental role in identifying key findings that help narrow down the differential diagnoses, define the extent of the disease, and contribute to the appropriate treatment of patients [2]. The treatment and prognosis of lesions vary depending on the etiology and range from conservative management to surgery and targeted cancer treatment [4].

The following case series presents neoplastic and non-neoplastic mesenteric lesions, some histopathologically confirmed, from the San Vicente Foundation Hospital in Medellín, Colombia, accompanied by a review of the literature emphasizing tomographic findings.

## Case presentation

Case 1

A 48-year-old male with neurofibromatosis type 2 (NF2) presented to the ED after blunt abdominal trauma from a traffic accident. Examination revealed abdominal wall hematomas but normal vital signs and no peritoneal irritation. Laboratory tests were normal, including complete blood count and acute phase reactants (Table 1).

**Table 1:** Laboratory results for each case are shown, with reference values in the last column. CRP, C-reactive protein; ESR, erythrocyte sedimentation rate.

Laboratory tests	Case 1	Case 2	Case 4	Case 5	Reference values
Hemoglobin	14.8	15	14.3	13	12-16 g/dL
Leukocytes	10.7	10.2	9.8	3.9	4.4-12 10^3^/µL
Lymphocytes	3.2	3	2.92	1.3	1.08-4.84 10^3^/µL
Neutrophils	6.6	6.1	5.75	2.4	1.5-7.26 10^3^/µL
Platelets	310	220	284	221	150-400 mil/mm^3^
CRP	0.87	0.8	4	9	<1 mg/dL
ESR	8	14	34	52	Males <15, females <20 mm/h

**Figure 1:**
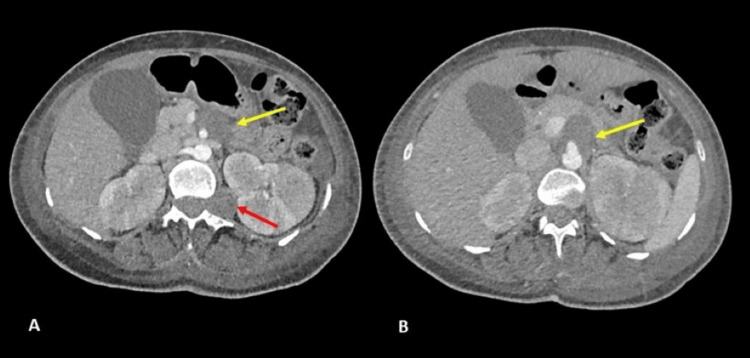
Contrast abdominal CT scan in arterial phase showing a mass (yellow arrow in A and B) with soft tissue density, without enhancement, and with well-defined borders, anterior to the celiac trunk. In addition, a lesion of similar characteristics of left paravertebral location, apparently in contact with the spinal canal and emerging from it (red arrow in A). Subsequently, the diagnosis of mesenteric schwannoma was made.

Contrast-enhanced abdominal CT, performed to exclude hemorrhage or intra-abdominal injuries, showed no traumatic injuries. However, well-defined soft tissue masses were identified in the mesentery (adjacent to the celiac trunk) and left paravertebral region (Figure 1).

The patient, previously asymptomatic, underwent percutaneous biopsy due to the lesions' non-specific appearance and his NF2 history to rule out a neoplasm. Biopsy confirmed schwannomas in the mesentery and paravertebral region (histopathological images are not available). The patient was discharged with follow-up indicated due to the benign nature of the lesions. 

Case 2

**Figure 2:**
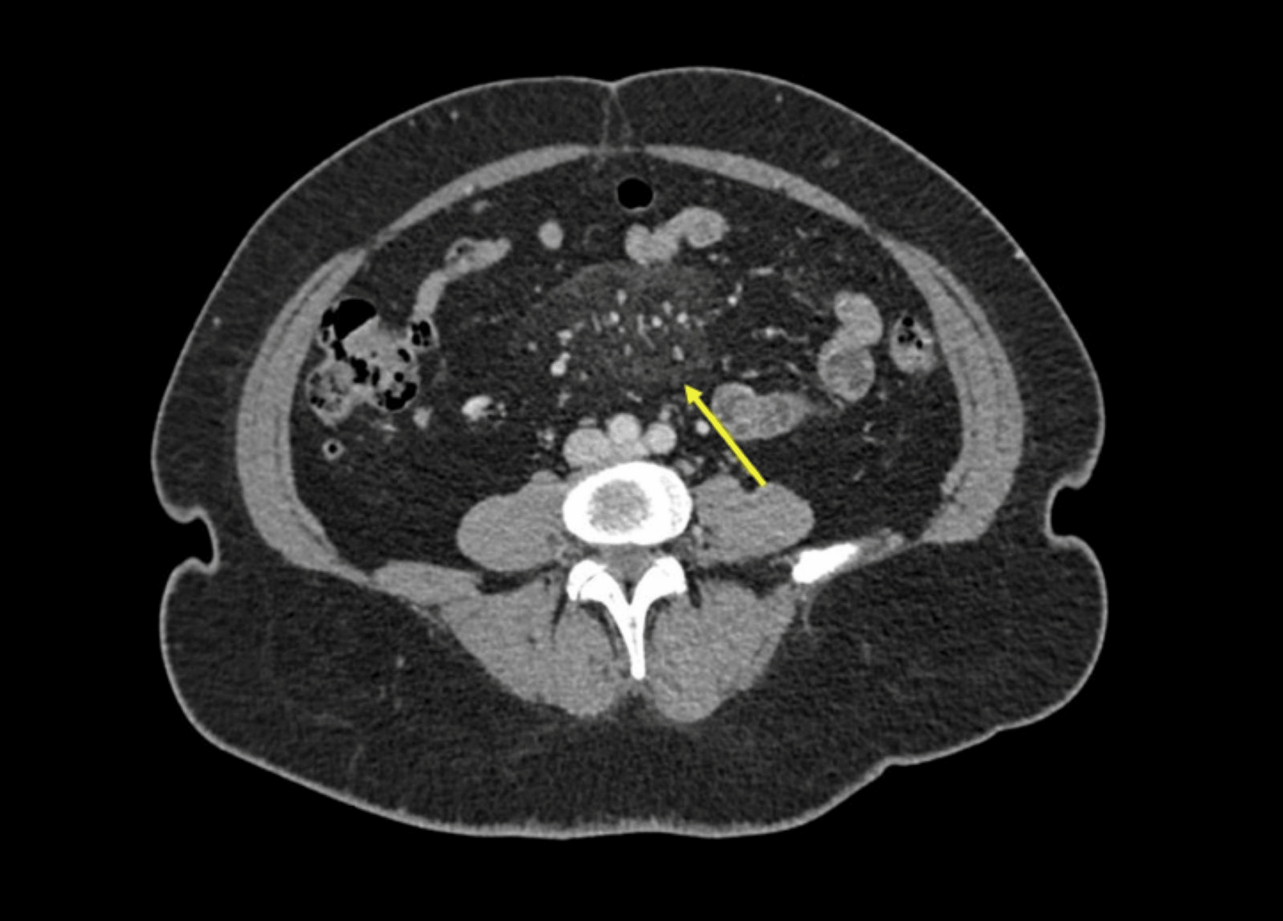
Contrast-enhanced abdominal CT scan showing increased density of mesenteric fat in the mesogastrium with some reactive lymph nodes (yellow arrow) due to mesenteric panniculitis.

A 32-year-old male patient with no significant medical history presented to the emergency department with a week of diffuse abdominal pain of non-specific characteristics and no fever or other associated symptoms. Examination revealed normal vital signs, no peritoneal signs, and mesogastric tenderness on deep palpation. Laboratory tests showed a normal CBC and acute phase reactants (Table 1). A contrast-enhanced abdominal CT scan was performed, revealing mesenteric fat stranding with sub-10 mm lymph nodes (Figure 2).

No other relevant findings were found on the CT scan, and a digestive endoscopy was performed without finding any abnormalities. Based on the above, a diagnosis of mesenteric panniculitis was made. The patient was treated with analgesics, resulting in complete resolution of pain, and was discharged with instructions to return if symptoms recurred.

Case 3

**Figure 3:**
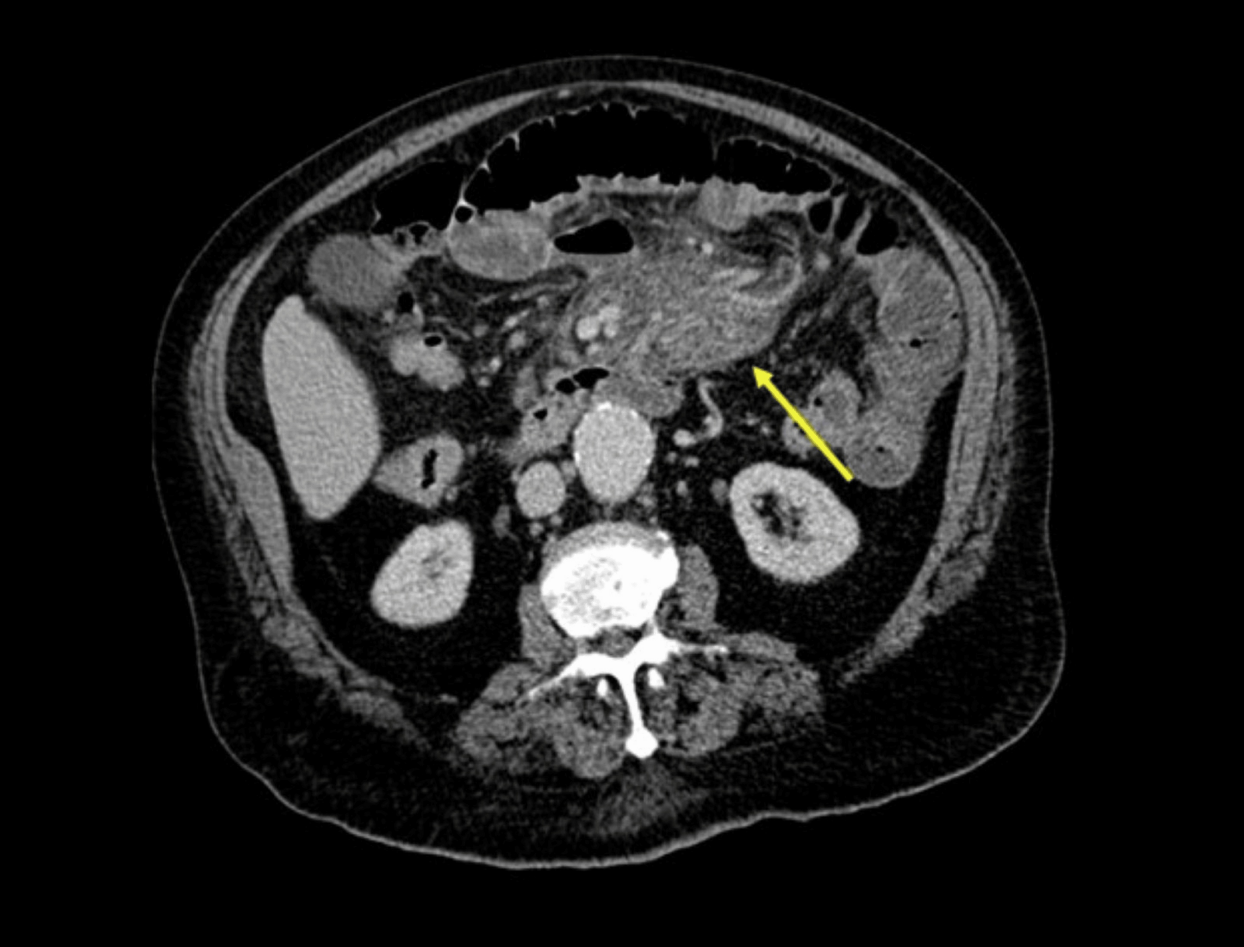
Contrast-enhanced abdominal CT scan in the portal phase showing increased density of mesenteric fat with a pseudomass appearance, engulfing the mesenteric vessels and extending to the adjacent intestinal loops (yellow arrow).

A 54-year-old male, in complete remission from Hodgkin's lymphoma (thoracic and abdominal regions) following chemotherapy two years prior, underwent a follow-up contrast-enhanced abdominal CT. The scan revealed increased density in the mesenteric fat, retracting mesenteric vessels, and extending to adjacent intestinal loops, resembling a pseudomass (Figure 3).

The patient was asymptomatic, with normal vital signs and no relevant findings on physical examination. Based on the imaging findings, physical examination, and history, sclerosing mesenteritis was suspected. It was decided to follow up with CT, showing no changes at one year of follow-up.

Case 4

**Figure 4:**
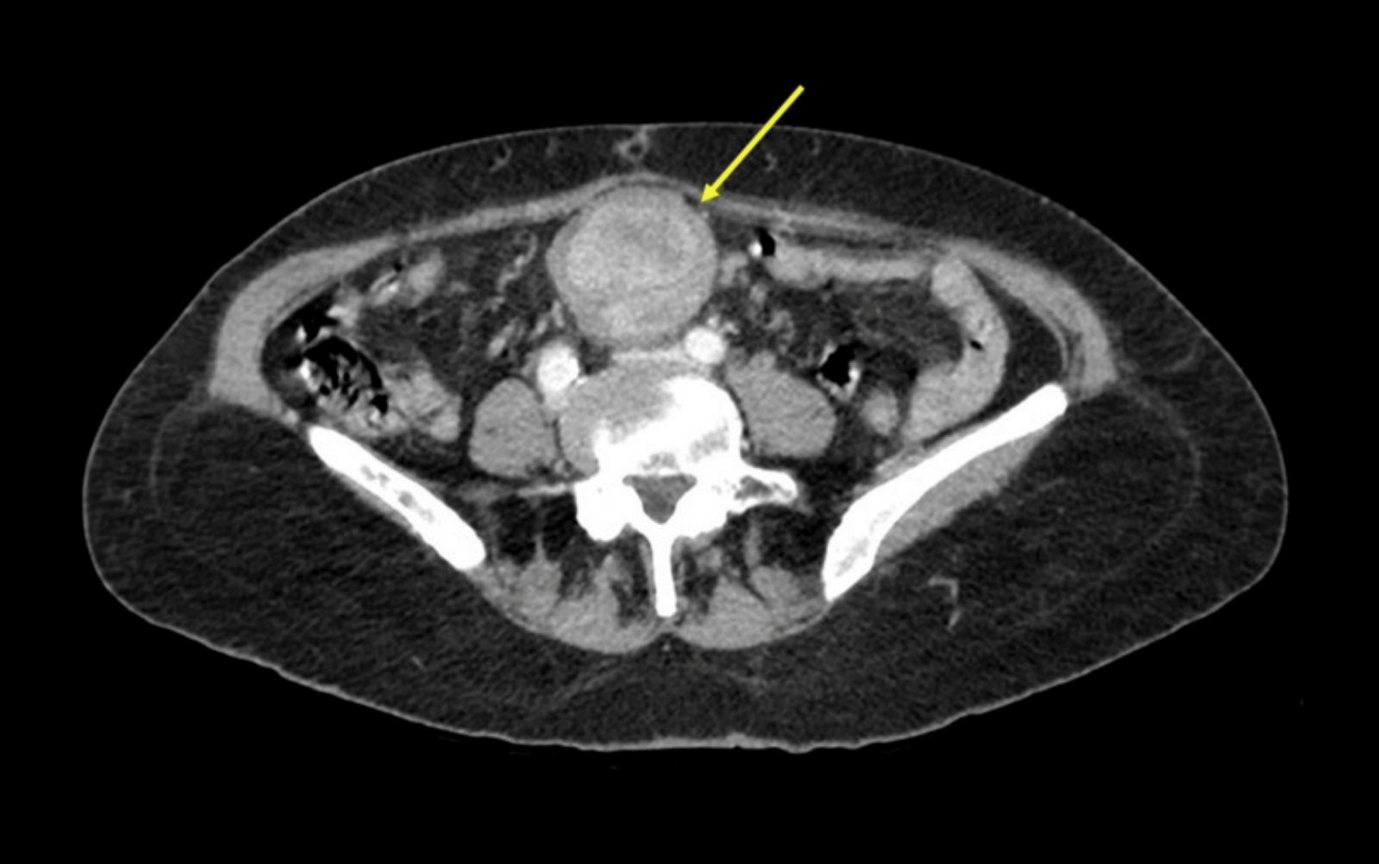
Contrast-enhanced abdominal CT scan showing a well-defined mesenteric mass with avid contrast enhancement (yellow arrow).

**Figure 5:**
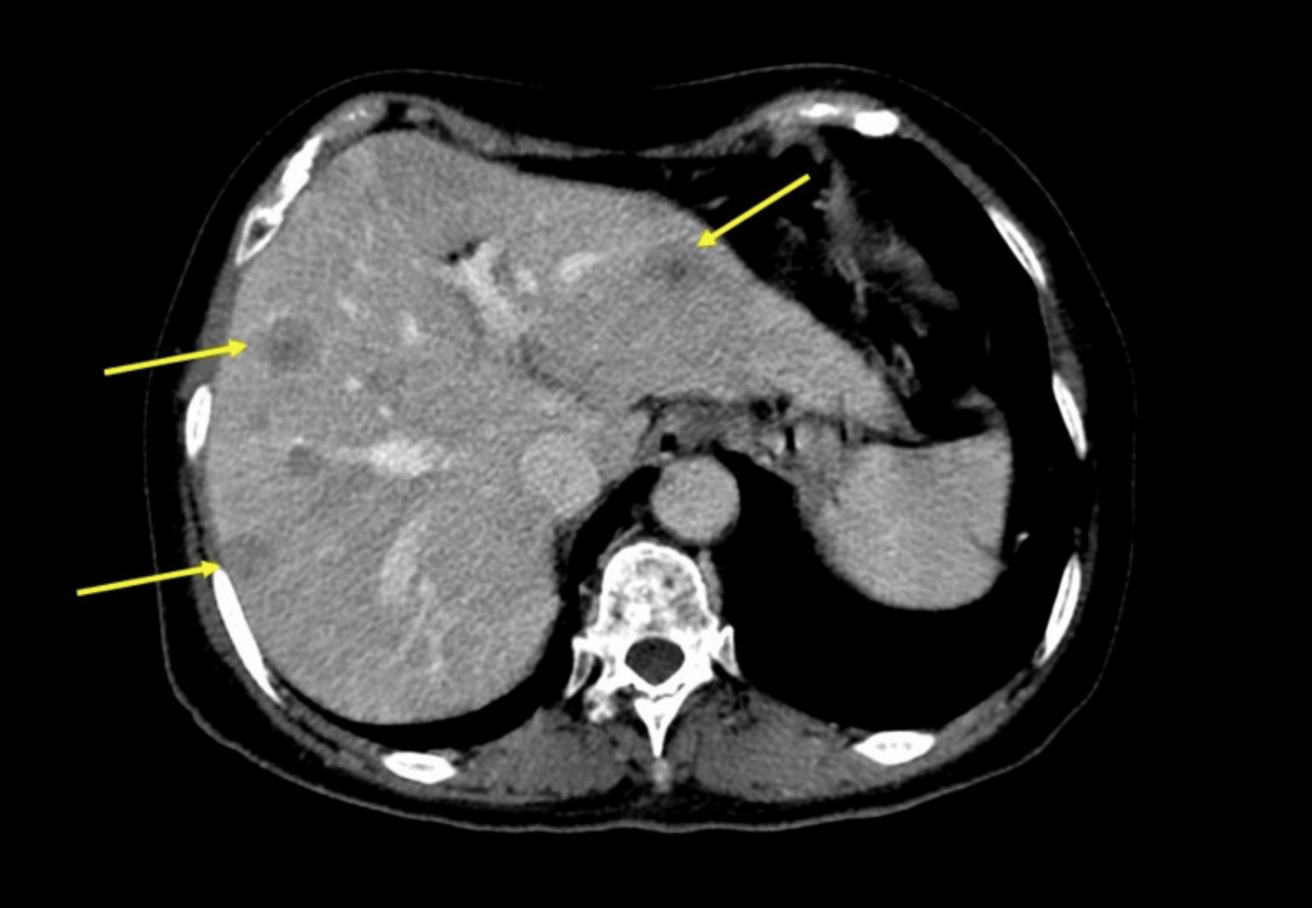
Contrast-enhanced abdominal CT scan showing multiple solid nodules in both hepatic lobes (yellow arrows) due to metastasis.

A 67-year-old female patient with no significant medical history presented with weight loss (approximately 5 kg in 3 months), intermittent mesogastric abdominal pain, and headache. Physical examination revealed normal vital signs, no peritoneal signs, and no other remarkable findings. Laboratory tests showed a normal blood count, with a slight elevation of acute phase reactants and normal renal function (Table 1). A contrast-enhanced abdominal CT scan was performed, showing a mesenteric mass with avid contrast enhancement and multiple metastatic lesions in the liver (Figures 4, 5).

A percutaneous biopsy of the mesenteric lesion and liver lesions was performed, confirming the diagnosis of neuroendocrine tumor (histopathological images are not available). No suspicious intestinal lesions were detected on the initial CT scan or on the subsequent gallium-68 PET/CT scan (Octreoscan®). The patient was treated with somatostatin analogs (octreotide), exhibiting good tolerance and progress.

Case 5

**Figure 6:**
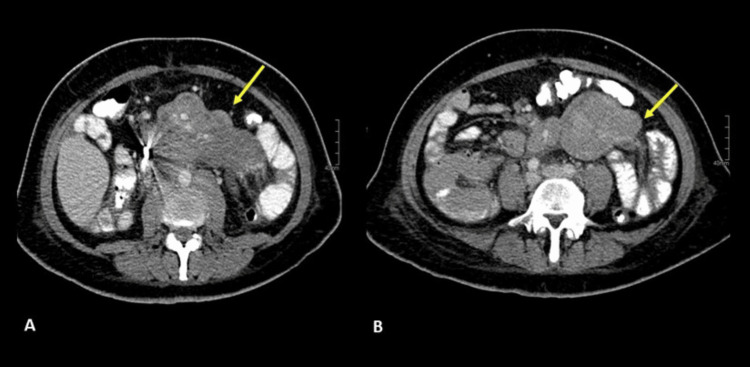
Contrast-enhanced abdominal CT scan showing confluent mesenteric, peri-celiac, and retroperitoneal lymphadenopathy (yellow arrows in A and B) surrounding the mesenteric vessels without invading them, with some central hypodense areas due to necrosis.

A 43-year-old female with no significant medical history presented with a three-month history of subjective weight loss, malaise, and night sweats. Physical examination revealed normal vital signs, pallor, and no other remarkable findings. Laboratory tests showed mild leukopenia and elevated acute phase reactants. A contrast-enhanced abdominal CT scan was performed, revealing multiple confluent adenopathies in the mesentery, surrounding the vascular structures without invading them; some adenopathies had slightly heterogeneous density, probably due to cystic degeneration (Figure 6).

A percutaneous biopsy of the lesions was performed, confirming the diagnosis of non-Hodgkin lymphoma (histopathological images are not available). Chemotherapy was started, but treatment has not yet been completed.

## Discussion

The mesentery is formed by a double layer of peritoneum that contains fat, vessels, lymphatics, and nerves. It comprises the true mesentery (small bowel mesentery and transverse and sigmoid mesocolon) and the specialized mesentery (lesser and greater omenta and mesoappendix). The true mesentery anchors the small intestine to the posterior abdominal wall, while the specialized mesentery connects multiple organs [1,2]. However, in radiologic literature, “the mesentery” typically refers to the true mesentery [2].

Primary mesenteric neoplasms are rare and often benign; secondary neoplasms are more common, arising from various intra- and extra-abdominal sites [1]. Benign non-neoplastic entities can mimic mesenteric tumors, including sclerosing mesenteritis and certain infections [1,2]. Differentiating these conditions is crucial for optimal treatment and requires a multimodal approach: thorough clinical examination, diagnostic imaging, and ultimately histology. Imaging, especially CT, plays a key role in narrowing differential diagnoses, defining disease extent, and guiding timely therapy [3].

Primary neoplasms

Most primary mesenteric neoplasms are mesenchymal and include lipomas, schwannomas, sarcomas, desmoid tumors, and neuroendocrine tumors (NETs). They are rare and must be confirmed histologically to distinguish them from secondary lesions [1-4].

Schwannomas are benign tumors from nerve coverings. They are slow-growing and may compress the originating nerve and nearby structures; mesenteric schwannomas are rare, often incidental, and occur mainly in younger to middle-aged adults, sometimes with NF2. On CT, they appear as well-defined, round-to-fusiform masses with variable density and often demonstrate heterogeneous enhancement due to cystic change or hemorrhage [4,5]. Imaging findings are non-specific, and histology is required for definitive diagnosis. Management ranges from observation to surgery depending on symptoms [5,6].

Non-neoplastic lesions

Benign non-neoplastic entities include sclerosing mesenteritis, mycobacterial infections, and other inflammatory or rare diseases [3,4].

Sclerosing mesenteritis is a rare, etiologically unclear condition that occurs most frequently in men (2:1) between the fifth and seventh decades of life [3,4,7]. It particularly affects the root of the mesentery and encompasses a spectrum of inflammation, fat necrosis, and fibrosis. Three histologic phases have been defined: lipodystrophy (predominance of fat necrosis), mesenteric panniculitis (predominance of inflammation), and sclerosing mesenteritis (predominance of fibrosis). Although the findings of these three phases may overlap in radiological images, the main findings in mesenteric panniculitis have been described as: increased attenuation of mesenteric fat (recognized in the literature as “the misty mesentery”) with pseudocapsule formation and occasionally with respect to the fat surrounding the vascular structures (fat ring sign) [3,4].

Retractile mesenteritis usually occurs in chronic processes, appearing on CT scans as masses with irregular contours and a spiculated appearance, which may have calcifications and tether adjacent bowel loops [3]. It is usually asymptomatic, though fibrosis can cause complications such as vessel thrombosis, hemorrhage, obstruction, or mesenteric ischemia [1]. Treatment is conservative for asymptomatic cases; surgery is reserved for refractory obstructive symptoms [3].

Mycobacterial infection (notably tuberculosis) can involve the GI tract, peritoneum, and mesentery. Mesenteric disease may be diffuse, with increased fat attenuation and nodularity, or present with necrotic adenopathy. Additional clues include ascites, smooth peritoneal thickening, and pulmonary tuberculosis findings [4].

Other rare inflammatory conditions affecting mesenteric lymph nodes include Whipple’s disease, celiac disease, Crohn’s disease, systemic mastocytosis, sarcoidosis, and Castleman’s disease [1-8].

Secondary neoplasms

Secondary mesenteric neoplasms are the most common and may originate intra-abdominally or from distant sites. They spread via direct, lymphatic, hematogenous, and peritoneal routes, often concurrently [1].

Direct Spread

Neoplasms that spread predominantly by direct invasion to the mesentery include NETs and gastric, pancreatobiliary, and colorectal primary neoplasms [1-4]. Of the above, NETs (or carcinoid tumors) of gastrointestinal origin are the most likely to spread directly to the mesentery. They are the most common malignant neoplasms of the small intestine but account for only 2% of gastrointestinal neoplasms [1,9]. Primary mesenteric NETs exist but are rare. Primary intestinal NETs originate in mucosal/submucosal neuroendocrine cells, with up to 40%-80% presenting with mesenteric involvement at diagnosis (directly and via lymphatics) [1]. The mesenteric lesion is usually the first to be discovered because the primary intestinal lesion is often small and difficult to visualize on conventional imaging. Mesenteric lesions typically appear as enhancing soft-tissue masses with surrounding fibrous bands extending to adjacent fat, often with a spiculated contour; calcifications occur in up to 70%, and nearby bowel wall thickening may be present [1-4]. Octreoscan® and Ga-68 PET-CT are the most appropriate studies for finding primary tumors that are not evident on tomography. Additionally, fluorodeoxyglucose PET-CT can be helpful in the diagnosis of undifferentiated tumors [3]. NETs range clinically from non-specific pain to carcinoid syndrome, commonly with liver metastases or in multiple endocrine neoplasia or type 1 neurofibromatosis [2,3]. Treatment spans from octreotide therapy to surgery, depending on symptoms [3].

Lymphatic Spread

Many neoplasms disseminate via lymphatics, with lymphoma being the most common mesenteric malignancy, followed by metastases from melanoma, colorectal, ovarian, breast, lung cancers, and others [1]. Mesenteric lymphoma is predominantly non-Hodgkin’s, though a minority are Hodgkin’s (30%-50% of non-Hodgkin's and 5% of Hodgkin's lymphoma) [2]. CT findings include: multiple rounded perivascular nodal masses, large heterogeneous masses displacing bowel, or infiltrative mesenteric fat involvement. Calcifications are uncommon at presentation but may appear after treatment [4]. Other metastatic neoplasms to the mesentery or inflammatory conditions may also involve the lymph nodes, but this involvement is usually more localized and smaller in size and number compared to lymphoma [1]. In addition, lymphoma is often accompanied by hepatosplenomegaly and retroperitoneal involvement [5].

Hematogenous Spread

Hematogenous spread occurs in melanomas, breast cancer, and lung cancer. Lesions may arise on the antimesenteric border of the small intestine, potentially serving as lead points for intussusception [1].

Peritoneal Spread

Peritoneal spread occurs with breast, gastric, pancreatic, and ovarian cancers, manifesting as localized mesenteric masses or diffuse mesenteric involvement (stellate mesentery). Peritoneal fluid dynamics often favor involvement in the lower right quadrant, frequently with peritoneal carcinomatosis [1,4]. Peritoneal mesothelioma can involve the mesentery as solid nodules or infiltrative disease, with nodules, peritoneal thickening, and ascites. A history of asbestos exposure and pleural disease supports the diagnosis [2,4,8].

The cases discussed reflect diverse primary and secondary mesenteric lesions, including rare primaries such as mesenteric schwannoma and mesenteric-origin NET, as well as non-neoplastic conditions (e.g., sclerosing mesenteritis) presenting with inflammatory and fibrotic patterns, highlighting imaging distinctions.

## Conclusions

Mesenteric neoplasms are rare pathologies. Most of them are metastatic, but they can rarely be primary, either benign or malignant. Some non-neoplastic pathologies can mimic tumors in tomographic images. Clinical presentation of both neoplastic and non-neoplastic lesions is variable, leading to low diagnostic suspicion, and there may be confusion with other pathologies. The definitive diagnosis usually requires histopathological analysis for confirmation; however, the role of tomography in conjunction with the clinical history is fundamental, identifying key findings that allow the differential diagnoses to be narrowed down and avoiding delays in the diagnostic approach and treatment. We present cases of neoplastic and non-neoplastic lesions of the mesentery, emphasizing the clinical and imaging characteristics that together helped to differentiate them.
